# A review of the applications of multi-agent reinforcement learning in smart factories

**DOI:** 10.3389/frobt.2022.1027340

**Published:** 2022-12-01

**Authors:** Fouad Bahrpeyma, Dirk Reichelt

**Affiliations:** Smart Production Systems, HTW Dresden, Dresden, Germany

**Keywords:** Industry 4.0, multi-agent reinforcement learning, smart factory, smart manufacturing, smart production systems, reinforcement learning, Artificial Intelligence

## Abstract

The smart factory is at the heart of Industry 4.0 and is the new paradigm for establishing advanced manufacturing systems and realizing modern manufacturing objectives such as mass customization, automation, efficiency, and self-organization all at once. Such manufacturing systems, however, are characterized by dynamic and complex environments where a large number of decisions should be made for smart components such as production machines and the material handling system in a real-time and optimal manner. AI offers key intelligent control approaches in order to realize efficiency, agility, and automation all at once. One of the most challenging problems faced in this regard is uncertainty, meaning that due to the dynamic nature of the smart manufacturing environments, sudden seen or unseen events occur that should be handled in real-time. Due to the complexity and high-dimensionality of smart factories, it is not possible to predict all the possible events or prepare appropriate scenarios to respond. Reinforcement learning is an AI technique that provides the intelligent control processes needed to deal with such uncertainties. Due to the distributed nature of smart factories and the presence of multiple decision-making components, multi-agent reinforcement learning (MARL) should be incorporated instead of single-agent reinforcement learning (SARL), which, due to the complexities involved in the development process, has attracted less attention. In this research, we will review the literature on the applications of MARL to tasks within a smart factory and then demonstrate a mapping connecting smart factory attributes to the equivalent MARL features, based on which we suggest MARL to be one of the most effective approaches for implementing the control mechanism for smart factories.

## 1 Introduction

As the market becomes more dynamic and complex and the global demand for small-batch, short-life products increases, manufacturing systems are facing more dynamic and complex environments. A smart factory, as the heart of Industry 4.0, is envisioned as a fully automated flexible manufacturing system consisting of highly connected smart components to provide mass customization and short product life cycles in a cost-effective, agile, and self-organizing manner. To move toward the realization of the concept of “smart factory”, manufacturing systems have been incrementally equipped with advanced enabling technologies such as (Industrial) Internet of things (I-IoT), cyber-physical systems, artificial intelligence (AI), cloud manufacturing systems, and big data technologies (Mittal et al., 2019). Taking these technologies into account as the backbones and building blocks of a smart factory, the key role, however, is played by the control mechanism that is responsible for making fine-grained and coarse-grained decisions to guarantee the performance of the manufacturing system. Simple examples of control mechanisms include the formation of robots, the design of paths between stations, the scheduling of operations, the management of inventories, the response to demands, and every decision that needs to be made to achieve specific goals (Jung et al., 2017). The control mechanism for smart factories, however, when compared with that of traditional manufacturing systems, is subject to various challenges. For the most part, high-level flexibility (required for smart factories) necessitates fine-grained decisions, thereby increasing the complexity of the control mechanism as more variables are introduced. This implies that the development of an optimal control mechanism will require the consideration of a larger number of contributing factors in the control equations, resulting in a longer engineering cycle and more complex equations. Traditional optimization approaches are often highly time-consuming and thus cannot be effectively incorporated to deal with the rapid and dynamic manufacturing environment in real-time. Furthermore, as manufacturing systems become larger in scale, the number of possible situations that need rapid and optimal responses will increase as well. As difficult as it is to predict all of the possible scenarios, what is even more challenging is determining the most effective reaction (real-time and efficient) to each case. Considering the complexity of this system and the limited accuracy and abilities of a human engineer, a detailed investigation of the potential situations and corresponding solutions is an extremely burdensome and expensive process. A smart factory offers a self-organizing solution to these situations through the incorporation of advanced intelligent control mechanisms, in which smart components make optimal decisions pseudo-independently in order to provide rapidity and can communicate with one another in order to act jointly toward global performance metrics such as efficiency, tardiness, and production rate.

According to the majority of the studies in the community such as Shi et al. (2020), Büchi et al. (2020), and Sjödin et al. (2018), in practice, realizing the concept of the smart factory in its full form is not possible without the use of advanced AI techniques. “Smart” in smart factories refers primarily to the application of artificial intelligence. AI provides powerful tools (for analytics and decision-making) for analyzing the vast amounts of data generated by smart components and for making optimal decisions. A variety of AI techniques have been used in the field to enhance automation, rapidity, and efficiency, such as neural networks (NNs) and their derivatives such as long short-term memory (LSTM) (Ozdemir and Koc, 2019; Nwakanma et al., 2021). These techniques were most commonly used to provide proactive decisions to pre-allocate optimal resources before the requests arrived. However, such approaches are limited when dealing with uncertainties such as machine breakdowns or the insertion of new jobs. A centralized control mechanism over multi-agent systems can also result in delays, and there is always a trade-off between global optimality and rapid response.

In this paper, *via* conducting a review study on the applications of MARL to decision-making problems within a smart factory, we suggest that one of the most effective solutions to realize self-organization in smart factories in an efficient and real-time manner can be found *via* multi-agent reinforcement learning (MARL), which offers a decentralized solution to dealing with uncertainty. MARL is an AI technique that incorporates reinforcement learning (RL) into multi-agent systems (MASs), where RL automatically finds optimal solutions to uncertainty through interactions between intelligent agents and the problem environment. More details on MARL are provided in Section 3. In order to demonstrate this, we will review the literature about MARL’s application to smart factory tasks and then discuss a mapping from smart factory requirements to MARL capabilities.

Several literature reviews of MARL approaches have been conducted in recent years, including those by OroojlooyJadid and Hajinezhad (2019), Nguyen et al. (2020), and Zhang et al. (2021), which were also used as the basis for this study. However, to the extent of our knowledge, this is the first study reviewing the applications of MARL into the smart factory tasks.

The remainder of this paper is organized as follows. In Section 1, we will describe the methodology, objectives, and boundaries of this research. Section 2 presents an overview of the RL and MARL systems. In Section 3, the literature is surveyed for the application of MARL approaches to tasks within a smart factory. Section 4 presents our conceptual analysis on the match between MARL and smart factory attributes. Section 5 provides a discussion on the concerns, limitations, and potentials of the applications of MARL to smart factory tasks. Finally, Section 6 concludes this paper and states the gaps and potentials for future research.

### 1.1 Research objectives, boundaries, and methodology

This research seeks to showcase the capabilities that MARL brings to realizing smart factories. To this end, this paper is mainly devoted to conducting a review study on the applications of MARL to the tasks within a smart factory. We will then draw a mapping from the required characteristics of smart factories to the corresponding capabilities in MARL. This paper provides practitioners and researchers in the field with an overall idea of how to address control problems in smart factories *via* the use of MARL. The size limitations of this paper, however, prevent attention to every detail since the ways of formulating tasks within the smart factory into MARL problems are extremely diverse and thus are beyond the scope of this study. In view of the fact that MARL is relatively new in the field of smart factories, this paper also discusses situations where RL is applied in conjunction with multi-agent systems in the relevant field, while focusing primarily on MARL. We mainly focus on cooperative approaches. Consequently, for each work, we briefly summarize the factors that indicate how MARL is integrated with the corresponding application.

## 2 Overview of multi-agent reinforcement learning approaches

This section provides a brief background on MARL. In this paper, when we refer to MARL, without the loss of generality, we address the case where RL is applied to or implemented *via* a multi-agent system. Therefore, we will consider a broader area than only the scope of MARL systems in theory. In essence, RL can be viewed as the most general form of the learning problems. Contrary to supervised machine learning, the target for an RL algorithm is a feedback which is partial and almost a delayed reward (or penalty). Moreover, RL differs from unsupervised learning, since its primary objective is to maximize reward signals rather than discover hidden structures within unlabeled data. Based on Sutton and Barto (1998), the ultimate objective of an RL system is the maximization of the expected value of the cumulative sum of a reward (immediate reward) signal. RL is generally aimed at achieving a long-term goal; therefore, the reward is back-propagated and discounted by a discount factor, and the goal is to maximize the discounted cumulative future rewards at a discounted rate.

**FIGURE 1:**
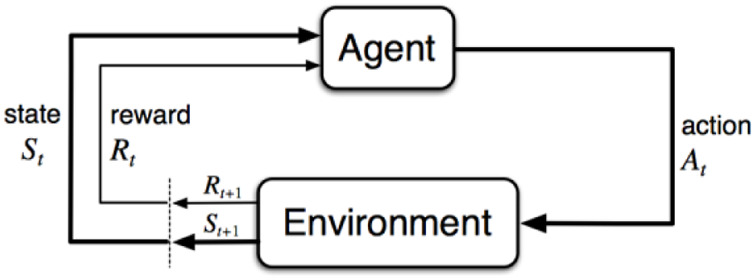
Agent in loop (Sutton and Barto (1998)).

RL problems are usually formalized using Markov decision processes (MDPs). MDPs are the mathematical representations of RL problems that originate in dynamic systems. The Markov property implies that all of the relevant information for a decision is encoded in the state vector *s*_*t*_. An MDP problem is described by the four-tuple M = [S; A; p; r], where *S* denotes the environment’s state space and *A* denotes the agent’s action space *a*_*t*_ ∈ *A*. In *s*_*t*_, the agent performs *a*_*t*_ in order to move to *s*_*t*+1_ and is immediately rewarded with *r*_*t*_. Additionally, *p*_*ss*_ represents the probability that a particular action performed at state *s*_*t*_ leads to the state *s*_*t*+1_ being reached. The uncertainty, however, is represented by a probability distribution function that indicates whether taking the action at the state *s*_*t*_ results in *s*_*t*+1_ for the agent. It emphasizes that the agent’s state transitions are partially influenced by the agent’s actions. Figure 1 illustrates the agent in an RL problem.

A more realistic version of MDP is the partially observable MDP (POMDP), wherein, as opposed to MDP, the agent only obtains a partial observation from the environment, leading to a higher level of uncertainty. For more details, please see Zhang et al. (2021).

The most popular RL techniques are Q-learning (QL), SARSA, and actor–critic (AC), each having variants as a means to enhance their performances in specific ways. Due to the limitations of this paper, here, we only briefly describe QL as the most popular RL technique and refer the reader to Arulkumaran et al., (2017) for other RL methods. In QL, the objective is to learn an action selection policy *π*_*t*_ (*a*|*s*) toward maximizing the reward. The policy *π*_*t*_ (*a*|*s*) represents the probability distribution that *a*_*t*_ = *a* if *s*_*t*_ = *s*. The Q-function *Q*: *S* × *A* → *R* yields the discounted cumulative reward with the discount factor *γ*.

$$Q\left(s,a\right)=r+\gamma \underset{a^{\prime }}{\max}Q\left(s^{\prime },a^{\prime }\right).$$ (1)

The QL algorithm is implemented to obtain the best action value function Q*(*s*, *a*), as shown in Eq. 2:

$$Q^{*}\left(s,a\right)=\underset{\pi }{\max}\;E\left[\underset{t}{\sum }r_{t}\gamma^{t}|s_{t}=s,a_{t}=a,\pi \right].$$ (2)

Q-values are updated using Eq. 3, every time an action is taken.

$$Q_{t+1}\left(s,a\right)=Q_{t}\left(s_{t},a_{t}\right)+\alpha \left(r_{t+1}+\gamma \underset{a}{\max}Q_{t}\left(s_{t+1},a\right)-Q_{t}\left(s_{t},a_{t}\right)\right).$$ (3)

With the emergence of deep neural networks (DNNs), the area of RL was able to address a wider range of applications due to generalization over past experiences for unseen situations, as opposed to traditional tabular RL, which was also vulnerable to the explosion of the state space due to the tabular formulation. DNNs are able to store values and approximate RL values for unseen situations. The well-known deep Q-network (DQN) and various types of AC methods rely upon DNNs. DNNs are one of the enabling technologies for MARL, since the extremely large state space of MARL problems cannot be contained in the tabular form.

In MARL, the actions taken by each agent change the environment and consequently change the perception of the other agents from the environment (the previous state before action *a* was taken is no longer valid, and the state has changed). This problem is known as non-stationarity.

**FIGURE 2:**
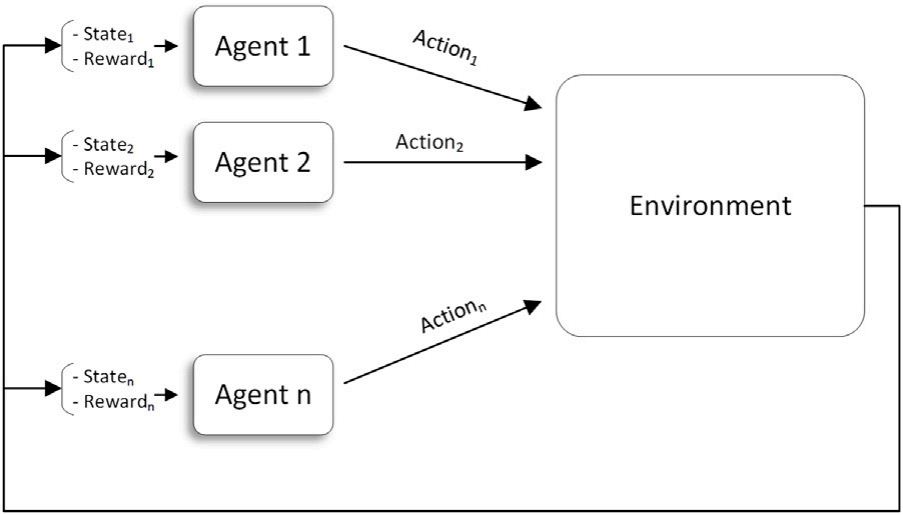
Agents in a multi-agent reinforcement learning (MARL) environment

MARL (Figure 2) incorporates multiple agents that interact with one another and with the environment. In accordance with the objectives of the application, this interaction may be cooperative, competitive, or mixed. This research focuses primarily on cooperative MARL, where the agents cooperate to maximize a common global reward while trying to maximize their own local rewards.

In the multi-agent setting, the generalization of MDP is the stochastic game. Based on Buşoniu et al. (2010), a stochastic game is described *via* a tuple (*X*, *U*_1_, …, *U*_*n*_, *f*, *ρ*_1_, …, *ρ*_*n*_), where *n* denotes the number of agents; *X* is the environment state space; and *U*_*i*_, *i* = 1, …, *n* represents the agents’ action space, leading to a joint action set ***U*** = *U*_1_ ×⋅⋅⋅× *U_n_*. The state transition probability function is defined as *f*: *X* × ***U*** × *X* → [0, 1], and the rewards for the agents are *ρ*_*i*_: *X* × ***U*** × *X* → *R*, *i* = 1, …, *n*.

The state transitions in the multi-agent case result from the joint actions of the agents at step *k*, $u_{k}={\left[u_{1,k}^{T},\ldots ,u_{n,k}^{T}\right]}^{T},u_{k}\in U,u_{i,k}\in U_{i}$ .

The transpose of a vector is indicated by T. The joint policy *h*_*i*_: *X* × *U*_*i*_ → [0, 1] represents all the policies. The joint action leads to the reward *r*_*i*,*k*+1_, and thus, the returns also depend on the joint policy.

$$R_{i}^{h}\left(x\right)=E\left\{\overset{\infty }{\underset{k=0}{\sum }}\gamma^{k}r_{i,k+1}|x_{0}=x,h\right\}.$$ (4)

MARL is mainly formalized *via* multi-agent (PO)MDP or decentralized (PO)MDP, which are referred to as M(PO)MDP and Dec-(PO)MDP, respectively. Depending on the application, the problem formulation may vary significantly, and thus, we refer the reader to Buşoniu et al. (2010) and Zhang et al. (2021) for more details and for the formal definition of MARL.

A variety of cooperative MARL approaches have been presented in the literature. The most famous methods are independent action learners (IALs), joint action learners (JALs), team-Q, distributed Q, distributed AC, communication-based, and network-based methods such as QMIX. Please see Zhang et al. (2021) for more details. We briefly describe some of the most popular MARL methods here.

**IAL**: Such as independent Q-learning (IQL), wherein agents take actions independently and only interact with each other through the environment. IAL methods incorporate RL individually and assume the other agents are part of the environment. These approaches, if not containing the impact of agents on each other, are subject to the problem of non-stationarity.

**FIGURE 3:**
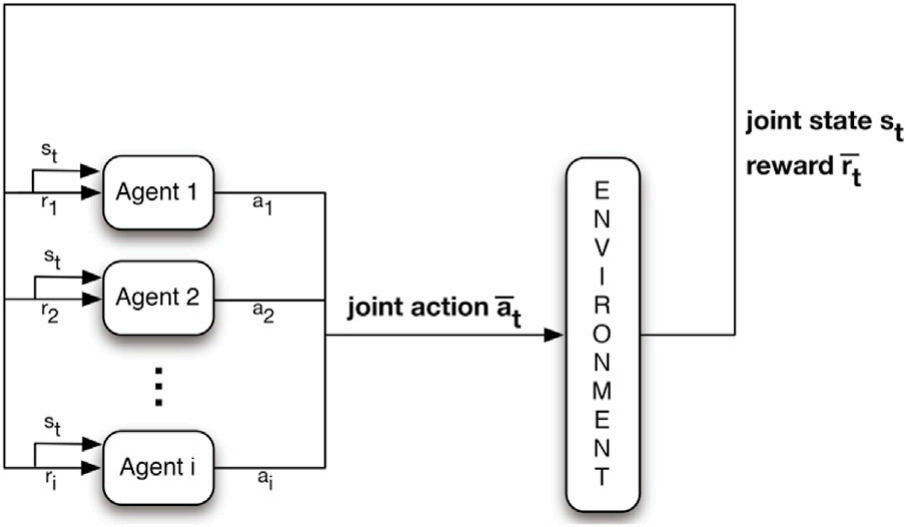
JAL (Nowé et al. (2012)).

**JAL** (Figure 3): Agents take (and learn the value of) joint actions to avoid non-stationarity. The most concerning challenge is, however, the credit assignment problem (identifying the agents’ individual share of the reward). In many cases, however, some agents become lazy in the team play because they do not identify their contribution to the global reward. In addition, the exploration strategy is difficult to design for JAL approaches since the joint action space for JAL is much larger than that of IAL.

**FIGURE 4:**
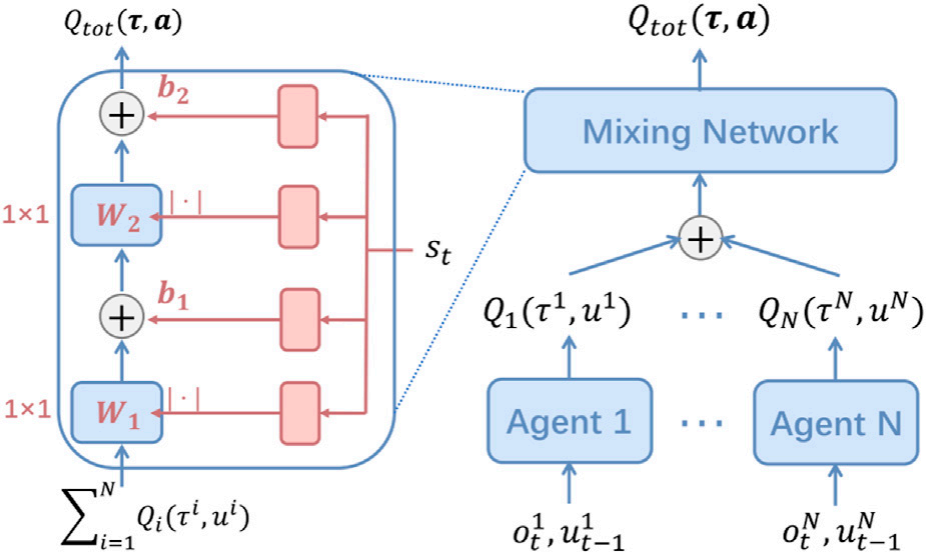
QMIX (Rashid et al. (2018)).

**QMIX** (Figure 4): QMIX serves as a representative value decomposition technique and works based on the principles of centralized training decentralized execution (CTDE). QMIX is primarily dependent on the mixing network to deal with the credit assignment problem. The mixing network receives the outputs of the individual agents’ Q-networks *Q*_*a*_ (*T*_*a*_, *u*_*a*_) to assign the individuals the credits and approximate the global Q-value *Q*_*tot*_ (*T*, *u*, *s*; *θ*). QMIX guarantees the individual-global maximum (IGM) principle, meaning that optimal local decisions jointly lead to the optimal joint decision for *Q*_*tot*_.

$$\underset{u}{argmax}\;Q_{\mathrm{tot}}\left(\tau ,u,s\right)=\left(\begin{array}{c} \underset{u^{1}}{argmax}\;Q_{1}\left(\tau^{1},u^{1}\right)\\ .\\ .\\ \underset{u^{n}}{argmax}\;Q_{n}\left(\tau^{n},u^{n}\right) \end{array}\right).$$ (5)

This enables the agents to take optimal local actions based on their local policies, while the joint action is also optimal for the entire system.

**FIGURE 5:**
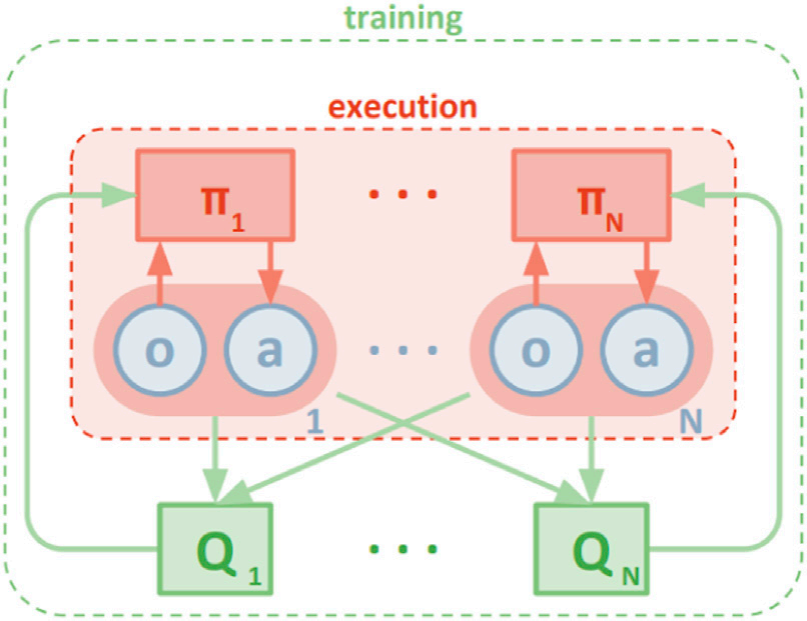
MA-DDPG (Lowe et al. (2017)).

**MADDPG** (Figure 5): Multi-agent DDPG suggests the association of each agent with a separate pair of actors and critics, training the critics centrally, and only using the actors during execution. Actors are trained by local state–action data, while critics receive training through the global state–action context.

## 3 Applications

There are various tasks within a smart factory that can be formulated as MARL problems, among which scheduling and transportation are of the highest popularity, while the literature has also paid attention to maintenance, energy management, and human–robot collaboration. In this paper, the emphasis is primarily placed on scheduling and transportation due to the large number of existing publications; other applications, due to limited attention in the literature, are discussed selectively to complete the study.

### 3.1 Scheduling

In the relevant literature, the job-shop scheduling problem (JSSP) and its variants have been extensively incorporated as abstractions of the manufacturing environment and smart manufacturing systems. Due to the JSSP’s NP-hard nature, only local optimal solutions can be obtained, which makes it difficult to address under multi-agent settings. Due to the complexity associated with developing such scheduling systems in dynamic environments such as smart factories, the use of self-adaptive and self-learning approaches in this regard has recently gained a great deal of attention from practitioners. MARL approaches offer varieties of advantages for developing such systems due to their effectiveness in dealing with uncertainties such as breakdowns and new job insertions.

We can categorize the related works concerning scheduling into centralized and decentralized classes.

#### 3.1.1 Centralized approaches

Centralized approaches consider a central controller that manages collaborations between agents, and as a result, this might lead to delayed decisions and an increase in computational complexity.

Gabel and Riedmiller (2008) proposed a tabular multi-agent QL approach for addressing dynamic scheduling problems in which unexpected events may occur, such as the arrival of new tasks or the breakdown of equipment, which would require frequent re-planning. Machine agents perform scheduling decisions based on local observations. A simple joint action selection method is used with tabular QL to provide reactive scheduling policies for dynamic scheduling environments.

Wang and Yan (2016) proposed a tabular multi-agent QL approach with joint action learners for adaptive assembly scheduling in an aero-engine manufacturing system. The authors use clustering to reduce the size of state space and assign fuzzy memberships to observations to link them to the states.

Sultana et al. (2020) proposed a multi-agent A2C approach to supply and chain management for multi-inventory systems in smart factories. The main goal of this work is to minimize under-stocking and over-stocking, where both impose negative impacts on the whole system. The warehouse is responsible for supplying multiple stores, which face requests for different types of products. This case is also linked to the smart factory concept, where, due to the mass customization feature of smart factories, demands can be submitted to any sub-factories, and thus, the product parts are collected from the corresponding store (that is placed inside or near the intended sub-factory). This work considers two types of agents: the warehouse and store agents. They both contribute to the accumulative reward and are trained *via* an A2C algorithm. The state vectors for both the warehouse and store agents consist of the demand forecast to further enhance the performance. The process of training is initiated by training the store agents independently with the full availability of stocks in the warehouse and then by training the warehouse agent based on the model obtained for the store agents. At the final step, the data are used to further train all the agents together for performance improvement. However, this work is more on the side of hierarchical reinforcement learning (HRL), and concerns such as non-stationarity have not been discussed and studied.

Luo et al. (2021a) presented a hierarchical RL (HRL) approach with two hierarchies for production scheduling in order to minimize the total tardiness and the average machine utilization rate. The paper uses a high-level DDQN agent to determine the global optimization goal, and a low-level DDQN is responsible for selecting appropriate dispatching rules. However, the use of a high-level controller agent in HRL approaches increases the depth of the RL problem and thus reduces the chance of convergence of the learning process.

Luo et al. (2021b) developed a hierarchical multi-agent proximal policy optimization (HMAPPO) approach as a means of dealing with the dynamic multi-objective flexible job-shop scheduling problem, where some operations are subject to the no-wait constraint. The method incorporates three types of agents, including the objective agent (as the controller), the job agent, and the machine agent (as the local actuators). The object agent periodically specifies temporary optimization objectives, the job agent chooses the job selection rule, and the machine agent chooses the machine assignment rules for the corresponding temporary objectives. With HRL, this method conducts learning at different levels of abstraction, whereby the high-level controller (the objective agent) learns policies over high-level objectives at a slow pace, while the lower-level actuators (job and machine agents) learn policies over low-level actions that meet the real-time constraints and goals. This means that some jobs should be continuously processed without interruption. However, with HRL, the architecture is not fully decentralized, as there should be a high-level controller at the top. It is also important to note that HRL approaches cannot guarantee the optimality of the overall aggregate policy of multiple agents.

#### 3.1.2 Decentralized approaches

As opposed to centralized approaches, decentralized methods are not characterized by a central control mechanism to manage agents toward their tasks. Decentralized approaches allow for agile decisions and a reduction in the overall computational complexities resulting from the elimination of the need for a central controller.

Qu et al. (2016) developed a two-agent Markov game approach based on QL to realize real-time cooperation between machines (scheduling) and the workforce (human resource management agents). This work aims to obtain an appropriate performance both for the scheduling agent and the human resource management agent for handling multi-process operations associated with different products in the dynamically changing environment of manufacturing systems.

Bouazza et al. (2017) presented a distributed QL approach for production scheduling that considers products as intelligent agents (which perform independently without a centralized control) and that intends to highlight the significant impact of considering the contribution of setup time (which is mainly neglected) in decision-making on the overall performance. Intelligent product agents can decompose decision-making into the choice of the machine selection rule and the selection of the dispatching rule. However, although agents each has impacts on the environment, this work fails to indicate the common impact of the decisions made by the agents toward the environment and toward each other.

Wang et al. (2017) developed a tabular multi-agent QL approach for dynamic flow-shop scheduling in manufacturing systems. Machine agents learn to make independent dispatching decisions simultaneously, regarding the expectations from other agents to deal with the changing environment. The proposed method showed superiority over first-in-first-out (FIFO), earliest due date (EDD), and shortest processing time (SPT) with respect to the mean flow time, mean lateness, and percentage of late jobs.

In Hong and Lee (2018), the authors used an asynchronous advantage actor–critic (A3C) to schedule some robotic arms for cluster cleaning in the semiconductor factory. The learning occurs in a two-stage process. In the first stage, the learning is carried out for the robot agent’s action selection policy *π*_*arm*_ (*a*_*R*_|*s*_*t*_ = *s*; *θ*_*arm*_), and in the second stage, the policy *π*_*i*,*j*_ (*a*_*i*,*j*_|*s*_*t*_ = *s*, *a*_*arm*_ = *a*_*R*_; *θ*_*i*,*j*_) is learned for deciding the start time of the cleaning operation in the *j* ^*th*^ chamber at the *i* ^*th*^ processing step. In order to perform MARL without dealing with non-stationarity, they used A3C, which enables learning in multiple parallel environments in an asynchronous manner.

In Waschneck et al. (2018a) (and also the extended experiments in Waschneck et al. (2018b)), the authors presented a multi-agent DQN approach for production scheduling in the semiconductor industry in an attempt to address re-entrant production flows and sequence-dependent setups. This work assigns agents to production stages and assigns jobs to a single machine in each stage. In an attempt to enhance stability, while all agents are trained independently, they use the DQNs of the other agents for the remainder of the work centers. While the environment is controlled under the influence of all the DQN agents associated with the agents, in order to deal with non-stationarity, only one agent trains its DQN actively at a time. The active agent considers the actions of the other agents during its training process. Due to the fact that all the agents strive toward maximizing a single global reward, the entire process can be described as cooperative reinforcement learning. The training process, in this work, comprises two phases. During the first phase, only one DQN agent is trained (separately repeated for each agent), while the rest are controlled using heuristics. The second phase consists of all work centers being controlled by DQN agents, while agents are trained in turn for a limited amount of time. The experiments indicate that the proposed method outperforms some dispatching heuristics. This work, however, appears to be incapable of handling changes in production requirements and the number of machines since the network must be retrained each time such changes are made.

Wang et al. (2018) proposed the concept of shared cognition *via* the use of a tabular multiple-agent QL to deal with disturbances in manufacturing cells. When disturbances occur, the corresponding agent distributes the information, and the agents share their cognition to raise the occurrence of a disturbance (a solution for that). Agents each incorporate a tabular QL model to learn the appropriate dynamic scheduling strategy without causing conflicts. The use of shared cognition provides the manufacturing cells with the ability to communicate and to distribute disturbed jobs to the appropriate cells in order to avoid conflicting with the quality of the service.

Motivated by the approach presented by Waschneck et al. (2018a), another multi-agent DQN-based approach was presented in Park et al. (2019) for production scheduling in semiconductor manufacturing systems. Regarding Waschneck et al. (2018a), the main objective in this work was to resolve issues with training the DQN agents in dealing with the scheduling problem under variable production requirements, such as a variable number of machines and a variable initial setup status. This work incorporates a shared DQN (presented in Foerster et al. (2016)) to improve the scheduling performance when dealing with variable production settings, especially the number of machines and the initial setup status. This approach, similar to Waschneck et al. (2018a), has two main phases. In the training phase, the production scheduling problem is practiced in an episodic manner *via* simulation. A double QL setting is incorporated, where a target DQN is used and updated periodically to resolve the stability issues encountered in the traditional DQN approach. Additionally, experience replay is considered to improve sample efficiency. In the second phase, the trained DQNs make appropriate scheduling decisions, even in unseen cases where some production parameters such as the number of machines and the initial setup times were not faced in the training phase.

Qu et al. (2019) developed a multi-agent AC approach for production scheduling that incorporates experts to guide the exploration of agents in order to improve the convergence of the distributed dynamic scheduling process in manufacturing systems. By following expert advice rather than randomly exploring the environment, the agents will be able to make more informed decisions at the start, leading to an increase in the speed of convergence. Agents select experts who perform better in the scheduling environment, observe their actions, and learn a scheduling policy from their demonstrations.

In Kim et al. (2020), the authors presented a multi-agent DQN-based approach that can learn from the dynamic environment and make better decisions regarding the allocation of jobs and the prioritization of tasks for mass customization. The DQN-based agents corresponding to different manufacturing components evaluate job priorities and schedule them *via* negotiation while continuously learning to improve their decision-making performance.

**FIGURE 6:**
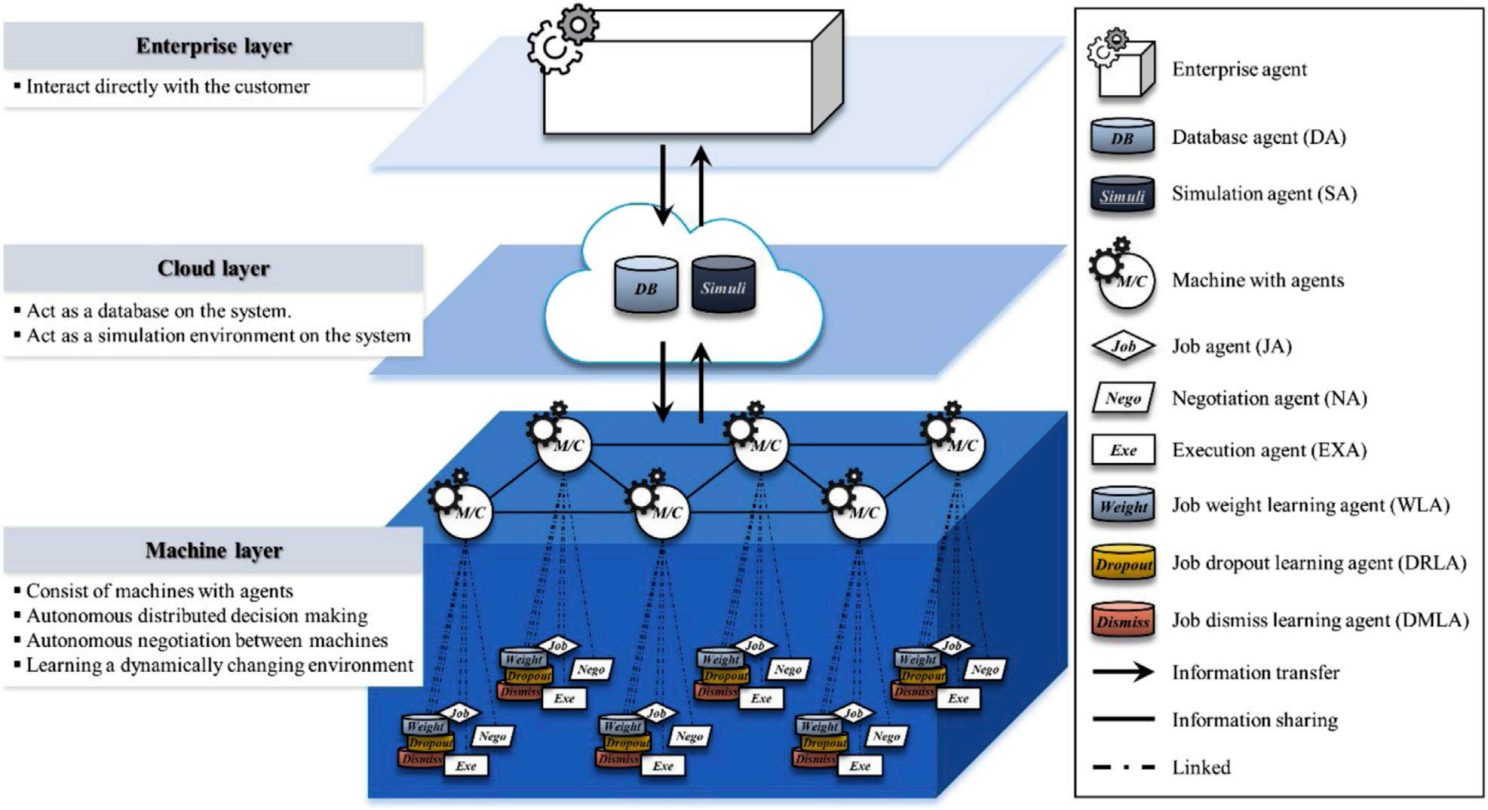
Smart factory presented in Kim et al. (2020).

The framework consists of three layers (enterprise, cloud, and machine layers). There is one enterprise agent (EA) associated with the enterprise layer and two agents associated with the cloud layer, namely, the database agent (DA) and the simulation agent (SA). The machine layer accommodates six types of intelligent agents, including job agents (JAs), negotiation agents (NAs), job weight learning agents (WLAs), job dropout learning agents (DRLAs), job dismiss learning agents (DMLAs), and execution agents (EXAs). These agents interact and cooperate to realize the following five types of functionalities: information sharing (*via* EA, DA, and SA), job index calculation (*via* JA), negotiation (*via* NA), learning (*via* WLA, SRLA, and DMLA), and execution (*via* EXA). This work is primarily centered on the use of RL for negotiation learning to realize communications between the agents in order to save the setup process. In this process, if the sum of the setup and performance times of incorporating more machines causes a longer job completion time than those of doing the job using fewer machines, negotiations will lead the scheduling process to use fewer machines. Figure 6 illustrates the architecture of the smart factory.

**FIGURE 7:**
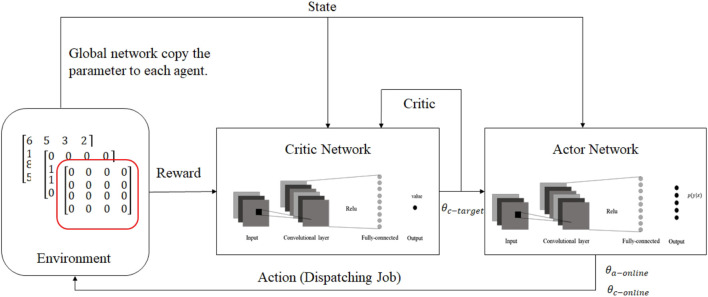
MARL method presented in Liu et al. (2020).

Liu et al. (2020) presented a parallel training mechanism under multi-agent settings by using a deep deterministic policy gradient (DDPG) approach and asynchronous updates in an effort to address the potential sources of uncertainty, such as machine breakdowns and unexpected incoming orders. In this work, machines are agents, and the state space includes a processing time matrix, a binary matrix of the jobs assigned to agents, and a binary matrix of completed jobs. These matrices are then used as inputs to a convolutional neural network (CNN), which is similar to the use of CNNs for RGB image processing tasks. The action space is limited to a choice among a set of dispatching rules, such as SPT and FIFO. Additionally, the reward is defined as a function of the process time of the selected job, the remaining process time of the job, and the comparison of the smallest makespan. Figure 7 represents the MARL approach presented in this work.

This work incorporates a global network that updates parameters based on the aggregate gradient of the exploring agents, and the exploring agents copy weights asynchronously from the global network. To deal with non-stationarity, agents are deployed in separate and parallel environments, each exploring a different part of the problem space, so that they cannot affect each other. Therefore, cooperation between agents under the MARL settings is established through the global network.

Wang (2020) developed a multi-agent weighted QL approach for adaptive job-shop scheduling. The work incorporates several agents, including machine, job, state, and buffer agents. These agents interact with each other to optimize earliness and tardiness in a dynamic manufacturing environment. This work uses the tabular QL form and works based on clustered system states and the degree of difference between the actual state and the cluster’s representative in order to avoid explosion of the state space. Actions are taken based on a search algorithm to obtain the maximum Q at the corresponding state (the cluster’s representative state). However, a negotiation protocol is used between the agents upon the receipt of a new job. A number of shortcomings of this approach can be identified, such as the need for a long initial exploration phase and the need to select appropriate parameters (for example, the number of clusters).

Baer et al. (2020) presented a multi-agent DQN that uses independent action learners through parameter sharing and an experience replay memory. Agents learn in turn, while the rest are fixed. Agents each incorporate a DQN with an input size equal to the state vector. Each agent consists of one DQN (in the parameter sharing approach) with an input layer for the length of the state vector. All scenarios have a fixed job specification and share an equal local optimization objective.

Dittrich and Fohlmeister (2020) proposed a DQN-based approach for order scheduling in manufacturing systems for minimizing the mean cycle time that implements MARL *via* the use of both local and global rewards (for achieving cooperation between agents). The machine agents only collect data, while the order agents use DQNs to make scheduling decisions. The agents communicate to exchange information and report statuses. This work incorporates the principles of CTDE. Local immediate rewards are given to order agents during the processing of orders, and global rewards redefine Q-values for a global DQN when the orders are complete and new orders are to be initiated. In other words, local DQNs are replaced by the global DQN when a new order is initiated.

In Denkena et al. (2021), the authors improved the work presented in Dittrich and Fohlmeister (2020) by incorporating the agent’s field of view and formulating the problem as a Dec-POMDP.

**FIGURE 8:**
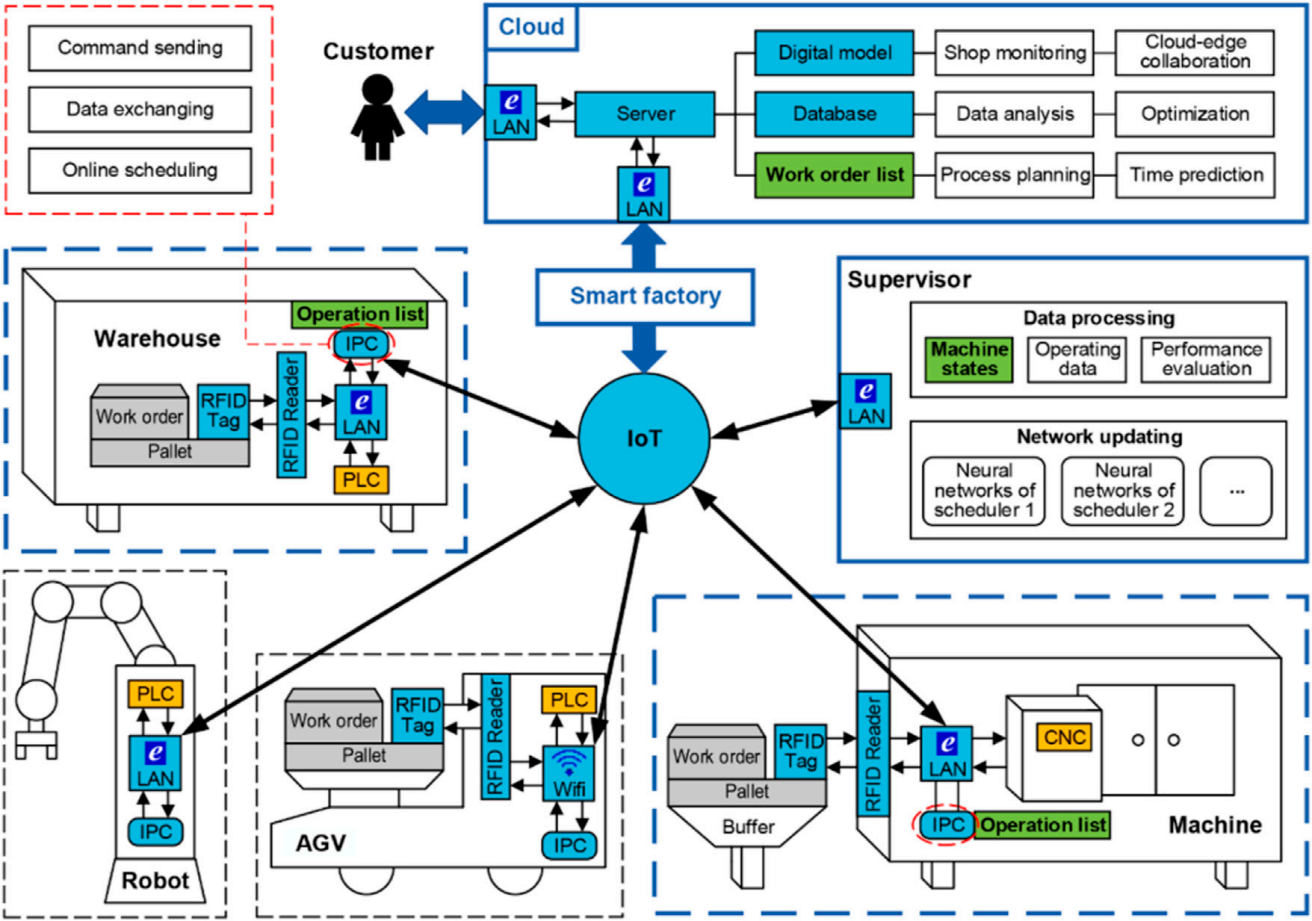
Smart factory presented in Zhou et al. (2021).

Zhou et al. (2021) proposed a smart factory comprising varieties of components and developed a multi-agent actor–critic approach for decentralized job scheduling. The machines each correspond with an actor–critic agent (enhanced also with target policy and target critic networks), which can all observe all the states of the other agents (*via* communication) in order to deal with the dynamic environment and also deal with non-stationarity. The architecture of the MARL approach for this work is illustrated in Figure 8. The schedulers or the actors in this paper are called the scheduling policy network, and they each schedule their next operation based on the states of the other machine. Thus, they make fully informed decisions.

Pol et al. (2021) addressed the challenge of achieving collaboration between multiple agents in MARL for scheduling purposes in manufacturing systems when dealing with objectives such as makespan minimization. The authors developed a handcrafted empirical logic to quickly estimate a reference makespan that works based on the sum of all operation times per job. They proposed a dense local reward augmented by global reward factors and a sparse global reward to realize cooperation between agents. Their work is based on DQN and simply includes other agents’ information in the state space of each agent to let them make more informed decisions when dealing with the dynamically changing environment of multiple agents. Communication is realized implicitly due to the fixed topology of the manufacturing system, and the DQN is shared among the agents to simplify the training process. To deal with the credit assignment problem, training is performed in two phases, that is, first with local rewards and then retaining with the local rewards augmented by a global reward factor. In addition, they proposed the use of sparse rewards given to each agent at the end of episodes, in place of the global reward, in an effort to simplify the process of learning to cooperate (by combining eligibility traces in place of replay memories).

Gankin et al. (2021) presented a multi-agent DQN approach for minimizing production costs in modular manufacturing systems. The environment comprises a grid of 5 × 5 production modules, and AGV units are used to carry products between the modules. AGV units are modeled as agents that use a shared DQN (with an experience buffer for training) to decide where to route jobs, considering the source module. Agents identify themselves based on their location, which is used in the state vector. Action filtering is considered *via* setting too low Q-values to invalidate actions. This work considers scheduling and routing simultaneously.

Wang et al. (2022b) incorporated the QMIX algorithm to develop a MARL approach for scheduling in resource preemption environments. QMIX works on CTDE, which provides agents with local and global objectives. QMIX has a mechanism that encourages the agents to achieve higher global rewards rather than only focusing on local rewards.

Johnson et al. (2022) proposed a multi-agent DQN approach for scheduling the assembly jobs that arrive dynamically in a robotic assembly cell. Their work is based on CTDE, with local independent agents and limited communications between agents to reduce communication costs. Agents each correspond to a robot that distributes jobs over a conveyor belt with a limited window size to workbenches. This work uses a shared DQN enriched by a target network to avoid the overestimation problem encountered with the standard multi-agent DQN architecture. The shared DQN is fed by properly encoded observation vectors to distinguish between the agents and equipped with a filter layer to filter out invalid actions in order to reduce the convergence time.

Gerpott et al. (2022) presented a distributed advantage actor–critic (A2C) method for production scheduling in two-stage hybrid flow-shop (THFS) manufacturing systems as an attempt to minimize the total tardiness and the makespan. This work uses completely identical scheduling agents that explore different parts of the problem space and share their gradients with the critic. This study uses global parameters that are shared by several agents who explore the environment concurrently. As a synchronous and deterministic method, A2C waits for each agent to complete the corresponding portion of experiments and then performs a global update by taking the average of all gradients received from the actors. A coordinator is used that manages the collection of local gradients and passes them to the global network.Table 1 summarizes the salient attributes of the reviewed applications of MARL to scheduling tasks within smart factories. In the table, the terms “Comm”, “Env”, “Col”, and “Dec” are the shortened forms for “communication quality”, “environment”, “collaboration method”, and “decentralized versus centralized management”, respectively. Collaboration quality represents the way in which collaboration is established between the agent, which can be through environment (Env), communication (Comm), parameter sharing (Param), state sharing (SS), a high-level controller (HLC), and asynchronous updates (ASUs).

**TABLE 1:** Comparison between the MARL approaches incorporated for scheduling in smart factories.

**Reference**	**RL method**	**Comm**	**Agent**	**Env**	**Metrics**	**Col**	**Dec/Cen**
Gabel and Riedmiller (2008)	QL	None	Scheduling agents	MMDP	Makespan	Env	Cen
Wang and Yan (2016)	QL	Explicit	Manufacturing cells	MDP	Run-through time and costs	Env	Cen
Sultana et al. (2020)	A2C	None	Warehouse and store	MDP	Replenishment cost	HLC	Cen
Luo et al. (2021a)	THDQN	None	Work-centers	MDP	Tardiness and utilization	HLC	Cen
Luo et al. (2021b)	HRL	Implicit	Objective, job, and machine agents	MDP	Tardiness, utilization rate, and workload variance	HLC	Cen
Qu et al. (2016)	QL	None	Machines	MDP	Total production costs	Env	Dec
Bouazza et al. (2017)	IQL	Limited	Products	MDP	Waiting time	Enc	Dec
Wang et al. (2017)	Tabular MAQL	None	Machines	MDP	(Mean) lateness, flow time, and late job rate	Env	Dec
Hong and Lee (2018)	A3C	None	Chamber and robots	MDP	Productivity	AsU	Dec
Waschneck et al. (2018a)	DQN	None	Work-centers	MDP	Run-through time and cycle times	Param	Dec
Waschneck et al. (2018b)	DQN	None	Work-centers	MDP	Capacity utilization	Env	Dec
Wang et al. (2018)	SMGWQL	Explicit	Manufacturing cell	MDP	Cost	Comm	Dec
Park et al. (2019)	DQN	Explicit	EA, DA, SA, NA, JA, WLA, DRLA, DMLA, and EXA	MDP	Makespan	Comm	Dec
Qu et al. (2019)	DQN	None	Scheduling agents	Semi-MDP	Work in progress and revenue	Param	Dec
Kim et al. (2020)	DQN	Explicit	Machines	MDP	Productivity and delay	Comm	Dec
Liu et al. (2020)	DDPG	None	Scheduling agents	MMDP	Makespan	AsU	Dec
Wang (2020)	WQ	Explicit	Job, state, machines, and buffers	MDP	Tardiness and run-time	Comm	Dec
Baer et al. (2020)	DQN	None	Independent schedulers	MDP	Makespan	Env	Dec
Dittrich and Fohlmeister (2020)	DQN	Explicit	Orders and machines	MDP	Mean cycle times	Param	Dec
Denkena et al. (2021)	DQN	Limited	Orders and machines	Dec-POMDP	Mean tardiness	Param	Dec
Zhou et al. (2021)	MAAC	Explicit	Machines	MDP	Makespan	Comm	Dec
Pol et al. (2021)	DQN	Implicit	Products	DEC-POMDP	Makespan	SS	Dec
Gankin et al. (2021)	MA-DQN	None	Workstations	MDP	Production rate	Param	Dec
Wang X. et al. (2022)	QMix	None	Jobs	DEC-POMDP	Makespan	QMix	Dec
Johnson et al. (2022)	DQN	Limited	Assembly cells	MDP	Makespan	Param	Dec
Gerpott et al. (2022)	A2C		Parallel scheduling agents	MDP	Tardiness and makespan	Param	Dec

### 3.2 Transportation and monitoring (moving agents)

Among the characteristics of smart factories is the large amount of work performed by autonomous moving devices serving a variety of functions, including material handling, monitoring, providing support, and delivering products. Moving robots such as drones, autonomous guided vehicles (AGVs), and overhead hoist transporters (OHTs) are able to move or transport material from one location to another, while the multi-agent setting imposes issues such as uncertainty. MARL has also been incorporated to address moving robots for various functions in smart factories.

#### 3.2.1 Multi-agent pathfinding

Pham et al. (2018) presented a multi-agent QL approach to UAV coordination for the optimal sensing coverage problem. In this work, UAVs are used as mobile sensors to provide visual coverage over a field. Thus, the aim is to coordinate UAVs in such a way that coverage is maximized and overlaps are minimized. The UAVs must then cooperate in order to accomplish the stated objective. A game-theoretical correlated equilibrium mechanism and a function approximation are used to address the challenges of joint-action selection and the high dimensions of the problem. The problem is simulated in a 3D environment of identical cubic cells and formulated as a general locational optimization problem. The joint-action selection problem requires that the agents reach a consensus, and thus the correlated equilibrium (which can be solved using linear programming) was used to evaluate the agreement regarding the selection of the joint-action set. This paper uses fixed sparse representation (FSR) and the radial basis function (RBF) as an attempt to map the original Q to a parameter vector *θ* by using state- and action-dependent basis functions *ϕ*.

In order to address the multi-agent path finding (MAPF) problem, Sartoretti et al. (2019) incorporated A3C to introduce a MARL approach called PRIMAL (pathfinding *via* reinforcement and imitation multi-agent learning). PRIMAL integrates RL with imitation learning (IL) to enable agents to learn the homogeneous path planning policy needed for online coordination in a POMDP environment. IL serves as a centralized planner and learns the impact of actions on the agents and the team as a whole in order to train the agents for coordination (behavioral cloning) in order to eliminate the need for explicit communication. PRIMAL was implemented in a partially observable discrete grid world with a limited field of view (FOV), meaning that the agents have local observations. Agents are modeled *via* a CNN of seven convolutional layers, followed by an LSTM, to approximate the individual agent’s policy. Collision results in a penalty, and a large positive reward is obtained upon the achievement of the goal. A3C trains the agents *via* local states, which might result in selfishness (locally optimized decisions), and thus, randomized environments were used to avoid selfishness. PRIMAL does not allow agents to follow the others. Thus, this leads to a reduced collision chance. More details on A3C can be found in Mnih et al. (2016).

Qie et al. (2019) proposed a multi-agent DDPG (MADDPG) approach for the multi-UAV task assignment and path planning problem. While the two tasks are optimization problems that are commonly addressed separately in dynamic environments, a large number of recalculations are required to be performed in real-time. In spite of the fact that all of the UAVs are identical, the formation of the UAVs over the distributed locations of the targets should be optimized to reach the total flight distance, taking into consideration the presence of risky areas in the field and the likelihood of collisions. During the training phase, all agents have access to the observations and actions of other agents through a distributed actor–critic architecture (DDPG uses actor–critic at its core). Actors see local observations, whereas critics have access to the entire observation space (each agent has an actor and a critic). During the execution phase, only actors are active in the field, which means that the execution process is decentralized.

Zhiyao and Sartoretti (2020) proposed *PRIMAL*_*c*_, which extended the PRIMAL’s search space from two dimensions to three dimensions. *PRIMAL*_*c*_ suggested the use of the capacity of agent modeling to enhance the performance of path planning *via* the prediction of the actions of other agents in a decoupled manner. However, learning others’ behaviors introduces the problem of prediction inaccuracies and would appear to be ineffective in practice.

Zhang et al. (2020b) proposed a decentralized multi-agent actor–critic-based framework that leverages the multi-step-ahead tree search (MATS) strategy to address the AGV pathfinding problem. To address scalability for a large number of agents while maintaining the response time within a predetermined range, experiments were conducted in a real-world warehouse. Different from PRIMAL (which avoids letting agents follow the others), this work allowed agents to follow the others to improve the job completion rate while avoiding collisions *via* the incorporation of MATS and post processing the actions. MATS assists in finding the possible actions from other agents so that possible collisions could be predicted and avoided by reducing the probability of taking actions that result in collisions. This work was able to outperform PRIMAL when applied to a real-world warehouse case.

Malus et al. (2020) incorporated the twin-delayed deep deterministic (TD3) policy gradient algorithm for autonomous mobile robots (AMRs) scheduling to address the complexities encountered due to rapid changes in the production environment and the tight relationships between dispatching and routing (planning and execution) problems. AMRs are distinguished from AGVs by their navigational capabilities. AMRs are equipped with sensory devices that detect the surrounding static and dynamic objects, allowing them to navigate and localize autonomously. Controlling a group of mobile robots is called a fleet management system (FMS), which performs tasks such as transportation, order dispatching, routing, and the scheduling of job executions. The problem with AMRs is that they are often tightly coupled, resulting in a system of immense computational complexity. Consequently, centralized approaches to managing fleets of AMRs often fail to provide real-time routing and dispatching at the same time. TD3 is an extension of the DDPG algorithm (both work on the basis of the actor–critic algorithm) used for continuous action-space problems wherein neural networks are incorporated to concurrently approximate two Q-networks and a policy network. In this work, with TD3, agents see local and partial observations, and each has a dedicated policy for mapping local states to actions. An agent’s action involves a bidding value between 0 and 1, where the highest value between the two currently assigned orders is taken (one for execution and one for the next processing step for real-time constraints). The reward design considers the cooperative nature of the agents, and all the agents receive the same reward/penalty. The order, once received by the agent, is added to the agent’s queue of orders, and the corresponding AMR vehicle performs it autonomously in a first-in-first-out manner. In the event that an order is completed within the time constraint, all agents receive a positive constant reward. Otherwise, they will be subjected to a penalty that increases quadratically with respect to tardiness.

Damani et al. (2021) later proposed PRIMAL2 to address lifelong multi-agent pathfinding (LMAPF) for applications such as smart warehousing in smart factories. LMAPF is a variant of MAPF, in which agents are assigned a new goal as soon as their current objective is reached in a dense and structured environment such as industrial warehouses. PRIMAL2 suggests the use of convention learning to enable the agents to learn a generalizable policy. Identifying certain conventions and forcing agents to learn them can enhance performance. They also incorporate environment randomization (sampling from a variety of environments during training) to enable the agents to learn to deal with different environments. Nevertheless, they assume that the tasks are sparsely distributed across random locations, thereby eliminating local congestion. PRIMAL2, like previous versions of PRIMAL, has a long training time.

Shen et al. (2021) combined the multi-agent asynchronous advantage actor–critic (MA-A3C) with an additional attention mechanism for the multi-AGV pick and place problem in smart warehousing systems. This attention mechanism allows attention to be focused on the beneficial information that arises from the interaction between AGV units in order to increase learning efficiency and minimize the corresponding complexity. They also used CTDE to address the dynamic Markov environment and non-stationarity. By incorporating the attention mechanism, this work is able to select AGV units dynamically during the training and thus improve the collaboration between AGV agents. The experiments were conducted on the Amazon Kiva system, consisting of a picking table, shelf, and AGV. Five possible choices are available in this system: up, down, right, left, and stay. The experiments reported that this method outperformed MAAC, MADDPG, MADDPG + SAC, and COMA + SAC.

Choi et al. (2022) incorporated QMIX for the cooperative control of AGVs in smart factories’ warehouses. An agent can choose an action in a grid-like environment (move forward, backward, to the left, to the right, and to stop) and receive an individual reward for its action. The reward is positive if the Manhattan distance to the target is reduced as a result of the action taken. CTDE was incorporated to address both the scalability issue of centralized learning and the non-stationarity of a fully decentralized learning process at the same time. The method is able to evaluate individual agents’ contributions due to the receipt of both the individual reward *Q*_*a*_ and the global reward *Q*_*tot*_ from the environment in QMIX. This method was able to outperform IQL.

Yun et al. (2022) incorporated an actor–critic method called CommNet for the deployment of CCTV-equipped multi-UAVs with a focus on autonomous network recovery to ensure reliable industry surveillance. As mobile CCTV UAVs can continuously move over a wide area, they provide a robust solution for surveillance in dynamic manufacturing environments. In order to enhance the surveillance performance, the study aims to improve the energy consumption of surveillance drones. In this case, surveillance drones are deployed in heavily populated areas. This work involves a single UAV serving as the communication leader, some UAVs serving as agents, and some targets serving as surveillance targets. Communication between UAV agents is handled by the leader UAV agent. The agents observe the local surroundings, take joint actions, and are rewarded individually and jointly for their cooperative efforts.

#### 3.2.2 Pathfinding + scheduling

Mukhutdinov et al. (2019) developed a multi-agent IQL approach for material handling in smart factories, inspired by the packet routing problem in computer networks. In this work, routing hubs are considered intelligent agents, which are equivalent to routers in computer networks. The formulation of this routing system is considered a graph, where nodes are router agents and edges are the paths between the agents. Each agent only observes its neighbor agents, and actions are defined as choosing between the outgoing edges. Each router agent has a DQN component for the approximating function *Q*_*v*_ (*S_v_*, *u*), which is the estimation of the minimal cost of the path from the routing agent *v* to the destination of the current node *d* *via* a neighbor *u*. A reward for action is the negated cost of the edge over which the packet has been sent: *r* = −*Cost* (*e*, *S_e_*). By modeling each router using a DQN, each router is able to account for heterogeneous data about its environment, which allows for the optimization of more complicated cost functions, such as the simultaneous optimization of bag delivery time and energy consumption in a baggage handling system.

Zhang et al. (2020a) proposed a centralized multi-agent DQN approach for the open-pit mining operational planning (OPMOP) problem (an NP-hard problem that seeks to balance the tradeoffs between mine productivity and operational costs), which works based on learning the memories from heterogeneous agents. Open-pit mine dispatch decisions coordinate the route planning of trucks to shovels and dumps for the loading and delivery of ore. The queuing of trucks as a result of a high truck arrival rate and the starvation of shovels as a result of a low truck arrival rate can both negatively impact productivity. Instead of being restricted to fixed routes, trucks can be dispatched to any shovel/dump in a dynamic allocation system. An appropriate dispatch policy should minimize both shovel starvation and truck queuing. This work presents an experience sharing DQN in order to provide a shared learning process for heterogeneous agents and also to deal with unplanned truck failures and the introduction of new trucks (without retraining). A post-processing step and a memory tailoring process were used to enable the DQNs to be trained by the samples obtained from trucks of diverse properties (heterogeneity).

Li et al. (2021) proposed MADDPG-IPF (information potential field) as a means of enhancing the adaptability of AGV coordination to different scenarios in smart factories for material handling. Typically, raw materials used in manufacturing workshops are stored at various locations throughout a warehouse. Thus, AGVs have to visit a number of locations in order to coordinate transportation tasks. To reach different targets, all AGVs must avoid collisions and self-organize as quickly as possible. The authors address the problem of reward sparsity *via* the incorporation of the information potential field (IPF) in the reward-shaping strategy, which brings stepwise rewards and implicitly leads AGVs toward material handling targets.

#### 3.2.3 Mobile operator

Karapantelakis and Fersman (2021) developed a multi-agent deep IQL-based approach to provide connectivity coverage services *via* distributed mobile network operators. To respond to the dynamic demands on mobile networks, mobile operators collaborate as individual agents. Agents have full observability of their environment and train their deep recurrent Q-network (DRQN) independently with respect to a common joint reward function. In order to mitigate the non-stationarity imposed by IQL, a cyclic replay memory (replacing old memories with recent ones) and a global target network are used.

#### 3.2.4 Overhead hoist transporters

Ahn and Park (2019) incorporated a graph neural network (GNN)-based factorized policy gradient (GNN-FPG) method based on the factorized actor-factorized critic (fAfC) method for the cooperative rebalancing of overhead hoist transporters (OHTs) as an attempt to enhance the productivity (reduce the lead, delivery, and retrieving times) of the material handling process in the semiconductor fabrication (Fab) system. OHTs are used to transport semiconductor wafers between machines and are considered to be an essential component of an automated material handling system (AMHS). Generally speaking, OHT refers to an automated transport system that travels on an overhead track *via* a belt-driven hoisting mechanism that facilitates direct access to the load port of the stocker. This work proposes a MARL algorithm for dispatching, routing, and rebalancing these OHTs. The problem of OHT rebalancing is quite similar to the problem of empty vehicle redistribution (EVR) in a traffic system. In this work, the Fab is discretized into a number of zones, and decentralized rebalancing strategies are developed for the idle OHTs of each zone (idle OHTs are assigned to new zones) in order to minimize the lead (retrieval) time and congestion. This work uses a collective decentralized partially observable Markov decision process (CDec-POMDP) for which the objective is to obtain a decentralized policy with respect to local observations in order to achieve the system-level goal. The proposed cooperative rebalancing strategy accepts the distributions of idle OHTs, working OHTs, and the loads (delivering tasks) over discretized zones in the Fab as an input and outputs decentralized rebalancing strategies for each zone.

Ahn and Park (2021) proposed a factorized actor–critic (FAC) method for establishing cooperative zone-based rebalancing (CZR) for OHTs in the semiconductor industry. The objective of this work is to reduce the average retrieval time and the OHT utilization ratio by incorporating graph neural networks. Using joint state and joint action information, a central model learns the interactions between the agents and the corresponding future accumulated shared return. Using only local observations and communication information, the learned policy is executed independently by the agent. The rebalancing problem is formulated as a partially observable Markov game (POMG), in which the Nash equilibrium policy of the game is to be determined. The agents in a stochastic game strive to obtain the policy that maximizes the expected accumulated reward. Decentralized optimal control was reformulated as a general stochastic game by the authors.

#### 3.2.5 Pick and place

Zong et al. (2022) proposed a multi-agent A2C approach for the cooperative pickup and delivery problem (as a variant of the vehicle routing problem) for warehousing in smart factories. The work also provides paired delivery, which implies that a vehicle might take more than one product part to deliver to more than one destination. In order to deal with the structural dependencies imposed between deliveries, this work incorporates a paired context embedding architecture based on the transformer model (Vaswani et al., 2017). A2C (with a joint critic and individual actors) and communication were used to build a centralized architecture. While different agents generate their individual policies (actors), they share the paired context embedding and context encoding within the centralized architecture of A2C.

Lau and Sengupta (2022) developed a shared experience actor–critic (SEAC) approach for the lifelong MAPF problem. The work was formulated as a partially observable Markov decision process, with the MARL’s aim being to determine the optimal joint policy of the agents. By sharing experiences, agents can learn from one another’s experiences without receiving the same rewards. In SEAC, the trajectories collected from other agents are incorporated for off-policy training, while importance sampling with a behavioral policy was used to correct the off-policy data.

Table 2 compares the literature in relation to the applications of MARL to transportation within smart factories in terms of its salient characteristics. In the table, the terms “Comm”, “Env”, “Col”, and “Dec” are the shortened forms for “communication quality”, “environment”, “collaboration method”, and “decentralized versus centralized management”, respectively. Collaboration quality represents the way in which collaboration is established between the agent, which can be through environment (Env), communication (Comm), parameter sharing (Param), state sharing (SS), a high-level controller (HLC), and asynchronous updates (ASUs).

**TABLE 2:** Comparison between the applications of MARL to transportation tasks in smart factories.

Reference	RL method	Comm	Agent	Env	Metrics	Col	Dec/Cen
Multi-agent pathfinding							
Pham et al. (2018)	MAQL	Implicit-limited	UAVs	Markov game	Coverage	Param	Cen
Sartoretti et al. (2019)	A3C	Implicit	Moving robots	POMDP	Travel distance and success rate	AsU	Dec
Qie et al. (2019)	MADDPG	None		MDP	Travel costs and collision	Param	Dec
Zhiyao and Sartoretti (2020)	A2C	Explicit	Moving robots	POMDP	Travel distance and success rate	AsU	Dec
Zhang Y. et al. (2020)	AC	None	AGV units	MDP	Collision rate and job completion rate	Param	Dec
Malus et al. (2020)	TD3	None	AMR agents	POMDP	Completion time	Param	Dec
Damani et al. (2021)	A3C	Implicit	Moving robots	POMDP	Travel distance and success rate	Param	Dec
Shen et al. (2021)	MAA3C	None	AGV units	MDP	Collision rate and travel distance	Comm	AsU
Choi et al. (2022)	QMix	Explicit	AGV units	Dec-POMDP	Path length and success rate	QMix	Dec
Yun et al. (2022)	AC	Explicit	UAVs	MMDP	Coverage	Comm	Dec
Multi-agent pathfinding	+scheduling						
Mukhutdinov et al. (2019)	IQL	None	Routing units	POMDP	Delivery time and energy	Env	Dec
Zhang C. et al. (2020)	EM-DQN	None	Trucks	POMDP	Production rate, cycle period, and matching factor	Param	Dec
Li et al. (2021)	MADDPG-IPF	Explicit	AGV units	POMDP	Task response time	Param	Dec
Mobile operator							
Karapantelakis and Fersman (2021)	IQL and DRQN	Explicit	Operator agents	Dec-POMPD	Service fulfillment	Env	Dec
OHTs							
Ahn and Park (2019)	fAfC	None	OHT zones	CDec-POMDP	Lead time and congestion	Param	Dec
Ahn and Park (2021)	Factorized AC	Explicit	Zone agents	POMG	Retrieval time and utilization ratio	Param	Dec
Pick and place							
Zong et al. (2022)	A2C (central critic)	Share info	AGV units	MDP	Travel distance	Param	Dec
Lau and Sengupta (2022)	SEAC	None	Vehicles	POMDP	Flow time, makespan, and delivery rate	Param	Dec

### 3.3 Maintenance

Considering various reasons such as machine breakdowns and deadlocks, smart factories also need automated and efficient maintenance strategies. MARL has been applied to maintenance problems in the relevant literature to provide a more automated solution to the uncertainty faced in the maintenance process. Wang et al. (2016) presented a tabular multi-agent QL approach for making maintenance decisions in a two-machine system. The presented method aims to make the agents learn the control-limit maintenance policy for each machine associated with the observed state represented by the yield level and buffer level. Due to the non-synchronicity of the state transitions between both machines, an asynchronous updating rule is also incorporated in the learning process. Zinn et al. (2021) presented a MARL system based on DQN and actor–critic to learn the distributed fault-tolerant control policies for automated production systems during fault recovery to increase availability. Liu et al. (2022) proposed a multi-agent DQN approach to make maintenance scheduling decisions for personnel and also production control during maintenance. This work incorporates a CNN-LSTM-based architecture for the DQN, while the impacts of agents are neglected. Su et al. (2022) presented a MARL approach using value decomposition actor–critic (VDAC) to enable physical machines (in a serial production line that requires multiple levels of machine decisions) to learn local maintenance policies in a distributed and cooperative manner. The proposed solution is formulated as a DEC-POMDP problem, and CTDE was used to provide the solution. Action masking is also incorporated to filter invalid actions. In VDAC, distributed actors make decisions for designated machines, while a central critic estimates the global state value.

### 3.4 Energy

The dependence of smart components in smart factories on electrical energy, together with the need for cost-effective, reliable, and efficient energy supplies, has led to the use of smart grids that are adaptive and can distribute energy in an on-demand manner. MARL has also been applied to smart grids. Smart grids and smart manufacturing systems share common properties/objectives, such as communication, integration, and automation, which define the commonalities of their applications. Samadi et al. (2020) presented a tabular MA-IQL approach for decentralized energy management in smart grids and proposed a system including high-level energy management agents, low-level heterogeneous resource agents, and consumer agents. The agents adapt themselves to maximize their profits without communication in the grid environment. Wang et al. (2022c) presented an MA-DDQN approach, named P-MADDQN, for resilience-driven routing and the scheduling of mobile energy storage systems (MESSs). This work formulates the problem of POMG and MESS agents interacting with the environment and making independent decisions for simultaneous routing and scheduling based on local information. Charbonnier et al. (2022) presented a tabular MA-QL approach for energy coordination management. Agents are proactive consumers, and local observations are modeled in a Dec-POMDP environment; they make individual decisions to find a trade-off between local, grid, and social objectives. Bollinger and Evins (2016) presented a MADQN and another MARL approach known as the continuous actor–critic learning automaton (CACLA) for optimizing technology deployment in distributed multi-energy systems. The work is based on technology agents, building agents, a grid agent, and a market agent. Alqahtani et al. (2022) presented a multi-agent AC approach for the energy scheduling and routing of a large fleet of electric vehicles (EVs) in a smart grid to address the power delivery problem. It is possible to use a fleet of EV batteries as a source of sustainable energy since they are capable of storing solar energy and discharging it to the power grids later on, which can result in lower energy costs. It is important to note that the use of a fleet of electric vehicles for power generation is only effective if they are dispersed appropriately across the area of need. The MARL approach is incorporated to address the joint problem of vehicle routing (VR) and energy dispatching (ED), with a special focus on enhancing the scalability and addressing the complexities involved. The problem is formulated as a DEC-MDP problem, while the vehicle’s position, the vehicle’s state of charge, solar irradiance, and power load are used as state variables. In addition, the action set includes mobility actions (up, down, left, right, or stay still) in the grid and energy dispatch decisions (charging, discharging, and idle). This paper uses actor–critic, where the actors are local and the critic is shared among the agents.

### 3.5 Human–robot collaboration

Humans play an important role in manufacturing systems, and their impact on the manufacturing environment is significant when designing smart factories. As a result, due to the unpredictable and dynamic behavior of human workers, their role should not be considered as a stationary part of the environment, while this fact has mainly been neglected in the relevant literature. HRC is a broad field, and the application of RL to HRC should be formulated in multi-agent settings. In this study, due to the limitations, we only bring some representative examples and leave the comprehensive study for future work.

Yu et al. (2021) presented a MA-DQN approach for scheduling human and robot collaborative tasks to optimize the completion time in an assembly chess board simulation of the manufacturing environment. The agent learns the optimal scheduling policy without the need for human intervention or expert knowledge, using a Markov game model. Wang et al. (2022a) incorporated a multi-agent extension of generative adversarial imitation learning (GAIL) to generate a diverse array of human behaviors from an example set. The behavior is then used in a MARL approach to account for the human during the human–robot handover and for the multi-step collaborative manipulation tasks. An approach presented by Zhang et al. (2022) generates the appropriate action sequence for humans and robots in collaborative assembly tasks using a MADDPG approach. A real-time display of the agent–human’s behavior is shown to the operator. In this scenario, the operator would be able to carry out the assembly task in accordance with the planned assembly behavior under the globally optimal strategy for the expected performance.

### 3.6 Other applications

Chen et al. (2021) presented a multi-agent DDPG approach for the coordinated welding of multiple robots with a continuous action space and local observations. Lan et al. (2021) studied the application of MARL to multi-robot pick and place problems in Dec-POMDP environments and suggested the use of the variants of MADQN and DRQN in combination with CTDE. Ji and Jin (2022) proposed a MARL system based on independent DQN agents in an attempt to capture self-organizing knowledge for developing multi-robot self-assembly systems. They obtained superior results for decentralized teamwork rather than a centralized approach.

### 3.7 Discussion and potentials

In this section, we review the applications that used MARL to address control problems in smart factories, with a main focus on scheduling and transportation and a brief review of maintenance, energy, and human–robot collaboration. Upon close examination, we can observe patterns for how MARL, which was a significantly difficult approach to implement, was able to be used for different tasks in smart factories. In this section, we identify a number of concerns and analyze them based on a review of the works provided earlier. The main concerns are the choice of the MARL method, scalability, problem formulation, scalability, convergence, a cooperation strategy, and information exchange. For the most part, MARL problems are significantly prone to non-stationarity, and different approaches showed certain ways to deal with this problem. Joint actions are one of the main approaches used to deal with non-stationarity, but this has drawbacks such as the difficulty of designing the exploration strategy due to the large joint action space. The other approach used was a profound support of agents, with all the necessary information about the states and actions of the other agents. However, the corresponding information sharing costs reduce the scalability and efficiency of this approach. A close look at the most comprehensive approaches that incorporate a larger number of agents, such as Kim et al. (2020) and Zhou et al. (2021), demonstrates the fact that scalable approaches tend to use more sparse communication strategies where agents are selectively chosen to exchange information to save time and cost. A more advanced approach, however, is the use of negotiation-based strategies incorporated in Wang (2020) and Kim et al. (2020), which can even be enhanced by taking advantage of negotiation learning. Furthermore, even those applications that used independent learners incorporated some techniques to deal with non-stationarity, such as asynchronous updates, CTDE, exploring separate parts of the environments in parallel with the aggregate gradient, and behavior forecasting. However, as the dynamicity of the environment grows, adequate information should be exchanged between agents for making informed decisions; otherwise, impacts from other agents on the environment cannot always be avoided or predicted. Therefore, we conclude that establishing spare communications is the recommended solution for both efficiency and rapidity. Collaboration is another important concern in MARL applications, which is mainly realized *via* the definition of a global reward. However, the credit assignment problem is challenging in this regard, since decomposing the reward to determine the share of each agent is not always possible. Without consideration of the credit assignment problem, it is possible that some of the agents become lazy and have a negative impact on the global performance of the system. Nonetheless, approaches such as QMIX suggest a dedicated approach to considering both global and local rewards in the process of learning. However, in real-time environments, QMIX might not show a good performance since an agent’s action might become dependent on the actions taken by other agents. The choice of the MARL method is also another concern, as it has an impact on the convergence and also on other performance factors. DQN, as the most popular approach, has certain limitations, such as the dimensionality and continuity of the state–action space. Altogether, AC-based methods such as MADDPG have other limitations such as slow convergence due to a larger number of parameters included in the learning process. As the last point to discuss in this section, the choice of state variables and the corresponding concept behind the definition of an agent have a significant impact on the performance of MARL systems. Considering the fact that the most successful applications of MARL incorporate supervised learning techniques such as NNs (and DNNs) for function approximation, the complexity of the problem grows significantly in the event of the choice of ineffective state variables. A correlation between these variables, randomness, anomalies, duplicates, low content, the irrelevance of a variable, and the number and usage of the variables, all influence the performance of the MARL system. As an example, the one hot encoding technique is one of the main approaches that should be incorporated when dealing with categorical state variables to be used as an input to a DNN.

## 4 Mapping from smart factory features to multi-agent reinforcement learning features

**FIGURE 9:**
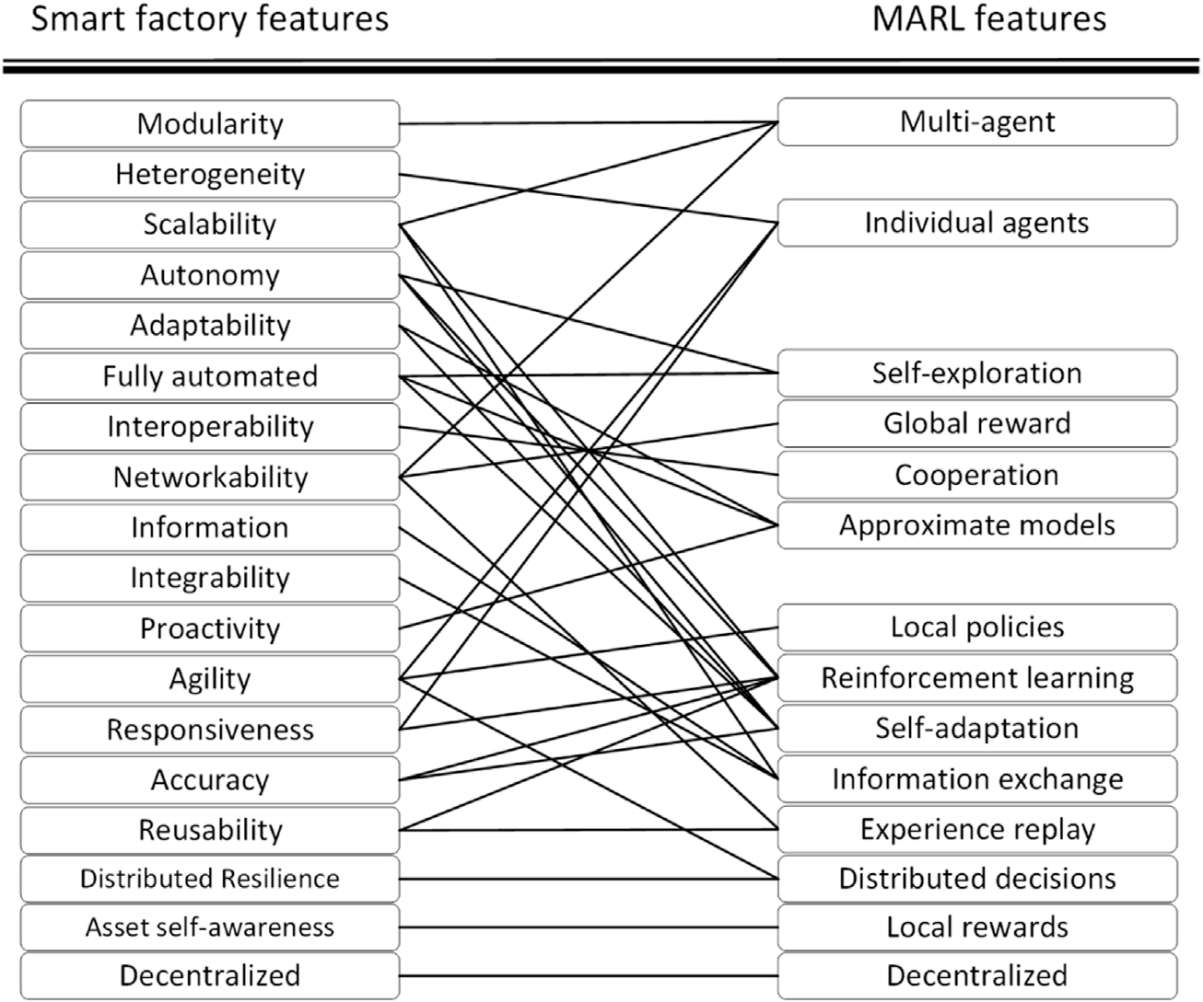
Matching between smart factory features and MARL features.

In the previous section, we reviewed the literature on the applications of MARL to tasks within a smart factory and noticed that there are various ways to convert these tasks into a MARL problem. As an early conclusion, considering the presence of uncertainty from various sources in the dynamic environment of smart factories, MARL has natural potential for dealing with uncertainty in smart factories in an automated manner. Many of the works reviewed in the previous section offer their solutions through POMDP problem environments that explicitly consider partial observability in their problem formulation, meaning that uncertainty is naturally considered with such applications. Furthermore, it is promising that MARL has a natural counterpart for almost all of the features required for establishing optimal management in smart factories, with all being provided at the same time and focusing on self-organization. Mittal et al. (2019) identified 22 main characteristics for smart factories, including digital presence, modularity, heterogeneity, scalability, context awareness, autonomy, adaptability, robustness, flexibility, fully automated, asset self-awareness, interoperability, networkability, information, appropriateness, integrability, sustainability, compositionality, composability, proactivity, reliability, agility, responsiveness, accuracy, reusability, decentralized, and distributed resilience. Regarding these features, focusing on the features that are explicitly important in the control mechanism for smart factories, we provide a match between the features required for a smart factory and the equivalent enabling features offered by MARL approaches, as shown in Figure 9.

**FIGURE 10:**
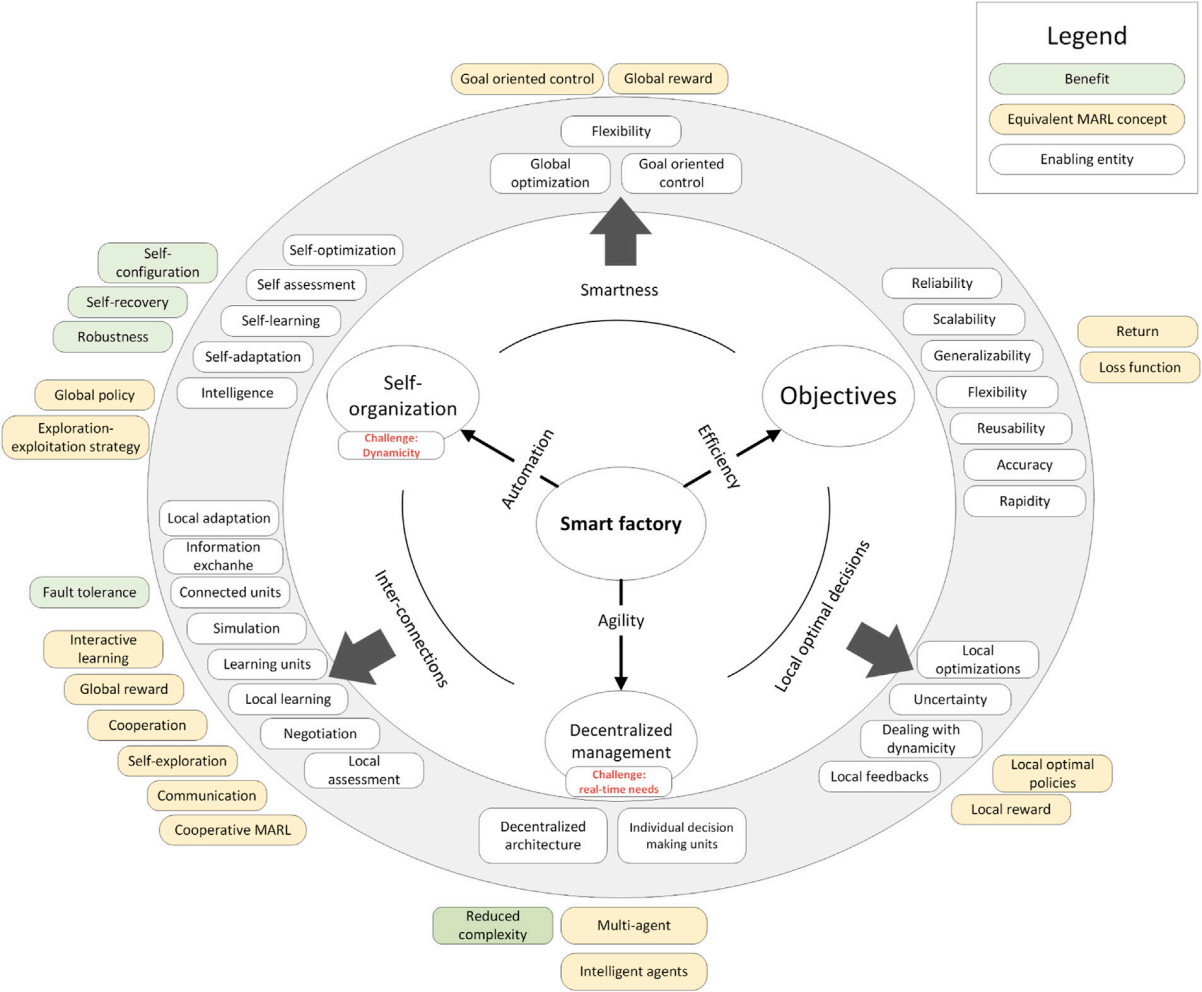
Mapping from smart factory features and MARL features.

Above all these characteristics, borrowed from Mittal et al. (2019), there are three leading factors in smart factories that are directly provided through the control mechanism, including automation, agility, and efficiency. Based on the review that we provided earlier in Section 3, we propose a mapping from the requirements of a smart factory initiated by these leading factors to the equivalent MARL features, shown in Figure 10.

As shown in Figure 10, the smart factory is mainly used to provide automation, efficiency, and agility. Agility is realized *via* decentralized decision-making by individual units, since the centralized form imposes delay as a result of the need for multi-objective optimization on a large number of factors and massive communications (regardless of the possibility of information loss) between the components. A decentralized architecture and individual decision-making units will both reduce the complexity of a manufacturing system. The equivalent concept with regard to these features of a smart factory is the multi-agent setting and the intelligent agents in a MARL framework. Automation at the highest level is provided *via* self-organization, where intelligence, self-centered assessment, optimization, learning, and adaptation are incorporated to deal with the dynamicity encountered in the manufacturing environment. These features, when established, can provide the manufacturing environment with the abilities of self-configuration, self-recovery, and robustness. The equivalent concepts in MARL for establishing these features are the global policy that controls the harmony of the entire system by defining the high-level long-term and short-term control directions for the low-level agents and the exploration–exploitation strategy that determines how and how often the system tries to explore novel behavior. Efficiency, as the main factor around which the entire solution is built, is highly dependent on the objectives defined by the application. Efficiency in practice is a multi-objective optimization concept, meaning that multiple factors contribute to the efficiency of the solution. The important characteristics that have a direct relationship with efficiency in smart factories are scalability, generalizability, flexibility, accuracy, and rapidity, while reliability and reusability can be considered as constraints when developing a control mechanism for a high-level or low-level task within a smart factory. Additionally, the equivalent concepts in a MARL framework, with regard to the mentioned objectives, are the return, which is the discounted cumulative future reward, and the loss function that appears in the training of deep neural networks used for complex MARL problems.

As shown in Figure 10, we have also identified the features that should be considered when each two of the three main factors are concerned. In order to not step beyond the scope of the study and also due to space limitations, we postpone the detailed analysis of these features to a future study.

## 5 Conclusion

In this study, we reviewed a wide range of applications that incorporated MARL into the tasks within a smart factory from a technical and analytical perspective. Specifically, MARL applications were studied for tasks including scheduling, transportation, maintenance, energy management, and human–robot collaboration, while the main focus was devoted to the first two categories. For the scheduling and transportation applications, we provided a comparative analysis representing how different MARL characteristics are chosen to implement the corresponding MARL solution. We also demonstrated how different aspects of smart factories match the objectives and capabilities of MARL and suggested a mapping from smart factory features to the equivalent concepts in MARL, indicating how MARL provides an appropriate solution to provide almost all the required features in the smart factory at once. Our investigations in this paper suggest that MARL is one of the most appropriate AI techniques for implementing tasks in smart factories, for the most part due to its natural ability to deal with uncertainty in multi-agent and decentralized systems, in a self-organized manner.
